# Pellagra Forum

**Published:** 1917-01

**Authors:** 


### PELLAGRA FORUM

*"In the Multitude of Counselors there is Safety."*

Address all communications on this subject to Dr. Martin.
Copy should be in Dr. Martin's hands by the 20th of the preceding month.

---

It has been mentioned by several writers and by many of the Southern physicians attending district associations, and especially was it remarked by many at the Atlanta meeting of the Southern Medical Association, that pellagra has decreased to a remarkable extent during the past year.

The government bulletins advocating a balanced diet and the general improvement in the financial condition of the farming classes were given credit for this remarkable decrease in new cases and also in the fatalities reported in various States.

While there is no doubt that better living means less pellagra, there is also another feature which must be considered.

Pellagra is a disease which only attacks the weak and those particularly non-resistant to its particular organism. During the past ten years the soil for pellagra has therefore been partly exhausted in many districts. It will probably be noted when all of the vital statistics are reported in the different States that the decrease began and was greatest in the States that first had pellagra.

It is to be hoped that better living and the survival of the fiittest will wipe pellagra out entirely in a few years. At present it is noteworthy that interest in the disease is waning and that we have had very few original articles on pellagra sent to the Forum for several months. E. H. M.

---

Do you know that a female fly lays an average of 120 eggs at a time?

---

"Are you of the opinion, James," asked a slim looking man of his companion, "than Dr. Smith's medicine does any good?"

"Not unless you follow the directions."

"What are the directions?"

"Keep the bottle tightly corked."—*Austin American.*

